# Co-creating an intervention to promote physical activity in adolescents with intellectual disabilities: lessons learned within the Move it, Move ID!-project

**DOI:** 10.1186/s40900-023-00420-x

**Published:** 2023-03-19

**Authors:** Laura Maenhout, Maïté Verloigne, Deborah Cairns, Greet Cardon, Geert Crombez, Craig Melville, Geert Van Hove, Sofie Compernolle

**Affiliations:** 1 Department of Movement and Sports Sciences, Ghent University, Watersportlaan 2, 9000 Ghent, Belgium; 2 Research Foundation Flanders (FWO), Brussels, Belgium; 3 Department of Public Health and Primary Care, Ghent University, Ghent, Belgium; 4 Institute of Health and Wellbeing, University of Glasgow, Glasgow, UK; 5 Department of Experimental-Clinical and Health Psychology, Ghent University, Ghent, Belgium; 6 Department of Special Needs Education, Ghent University, Ghent, Belgium

**Keywords:** Co-creation, Intervention, Physical activity, Adolescents, Intellectual disabilities, Patient and public involvement

## Abstract

**Background:**

Co-creation is a method to develop acceptable, contextually appropriate and potentially more effective interventions. Adolescents with intellectual disabilities (ID) seldomly participate in research and program development due to the assumption that they lack the capacity to understand and discuss the related topics.

**Objective:**

This study describes reflections on a co-creation process with adolescents with ID from the point of view of the researchers in developing an intervention to increase physical activity. It was the aim to highlight elements that must be considered when implementing co-creation and consequently formulate important lessons learned.

**Methods:**

Twenty-three adolescents (14–22 y) with mild to moderate ID participated in six co-creation sessions at their school. The objectives and working methods in each session are described. Inductive thematic analysis was conducted on the researchers' reflection forms, which were completed after each session.

**Results:**

Seven main themes could be distinguished from the data: experiences related to assistance (i.e., teacher presence) during sessions, the importance of building rapport, co-decision making power, the impact of different group dynamics, the relevance of adapted questioning, the influence of co-creative working methods and required characteristics of a co-creation researcher.

**Conclusion:**

Seven lessons learned were formulated when preparing and conducting co-creation with adolescents with ID. Innovative, concrete (non-abstract) and creative working methods are highly needed. Describing the entire process transparently could be a first step to turn co-creative research into an evidence-based methodology.

**Supplementary Information:**

The online version contains supplementary material available at 10.1186/s40900-023-00420-x.

## Abstract (summary)

Studies show that people with intellectual disabilities are less physically active than the general population. This is a problem, since people with intellectual disabilities experience more health problems, and physical activity might be an important angle to reduce these health problems. However, current interventions to promote physical activity in this target group do not appear to work because they do not match their needs and preferences. Therefore, it is important to develop interventions together with them, in collaboration, what is called "co-creation”. This has not happened much in research with people with intellectual disabilities before (and especially not with adolescents having intellectual disabilities), because researchers often have the perception that they do not have the capabilities to understand and discuss research related topics. This study elaborates on the researchers’ experiences in conducting co-creative research with adolescents and young adults with mild to moderate intellectual disabilities, and formulates 'lessons learned' so that future researchers can start from these findings when they themselves want to engage in a co-creation process with this target group. The results showed that co-creation is feasible with this target group, if co-creation methods are selected that fit the target group (e.g. making use of visuals, asking concrete (non-abstract) questions and providing clear but short instructions). We suggest that (standardized) innovative and creative working methods are needed when conducting co-creation with this target group. Moreover, to be better armed against the enormous flexibility expected of a co-creative researcher, it might be helpful to make an assessment of the group dynamics before conducting co-creation. The presence and contribution of the physical education teacher in these co-creation sessions was seen as an added value for several reasons. By describing this entire process transparently and in detail, this could be a first step in making co-creation an evidence-based methodology, also for vulnerable populations.

**Supplementary Information:**

The online version contains supplementary material available at 10.1186/s40900-023-00420-x.

## Background

Intellectual disabilities (ID) are defined as limitations in intellectual functioning (intelligence quotient (IQ) < 70) and adaptive behaviour, with an onset in childhood (< 22 years) [1]. Evidence shows that people with ID face more health problems than their peers without ID, such as higher rates of obesity, constipation, cardiovascular diseases and mental health problems [2–5]. This results in a reduction of about 20 years of life expectancy in comparison to the general population [6]. It is clear that these health inequalities, affecting an estimated 1–3% of people worldwide [7, 8], must be urgently addressed. One way is to promote a healthy lifestyle in young people with ID, such as the increase of physical activity (PA) [6, 9]. PA is associated with improved physical and mental health among adolescents [10–15]. However, adolescents with ID are less physically active than their typically developing peers [16–21]. Low activity levels furthermore track into adulthood, as adults with ID have also been reported to participate in little or no PA [22, 23]. Despite the need to find ways to promote PA in adolescents with ID [15, 16, 24–26], they are a neglected population in PA research. A systematic review from 2018 showed that only five studies involved adolescents with ID as participants in PA promotion interventions [16]. Moreover, the interventions were found to be mostly ineffective (i.e., 4/5).

A reason for this ineffectiveness could be the lack of a population-specific approach in the development of the interventions [16, 27]. Adolescents with ID do not connect to PA interventions for the general population because the specific interpretation of influencing PA factors (e.g., social support) among these adolescents differ from those of their peers without ID [19, 20, 28, 29]. It is consequently key to immerse oneself in the world of this target group and look for tailored ways to promote their PA specifically. A co-creational approach is therefore seen as promising for the development of more acceptable, contextually appropriate and potentially more effective interventions [30–42]. Although co-creation is used in many domains (e.g. marketing), the definition of co-creation for public health interventions is used within this paper, which is *collaborative public health intervention development by academics working alongside other stakeholders* [32, 39], in which the population of interest is one of the most important stakeholders. Co-creation is supported by UN’s Sustainable Development Goals (SDG #17, ‘Partnership for the Goals’) as a necessary approach to reach public values such as citizenship, social justice and well-being [40, 43] and seems especially valuable to learn about and work with vulnerable, disadvantaged or at-risk populations, such as people with ID [31, 44, 45]. Moreover, co-creation seeks to centralise participation, which is one of the corner stones of the International Classification of Functioning, Disability and Health (ICF) framework [46]. Unfortunately, individuals with ID are seldom invited to express their opinions and emotions in research (e.g., on PA promotion interventions) [47]. Parents or professional caregivers are often interviewed on their behalf. This can result in the carers’ views being presented rather than the true participant preferences or experiences. This not only has implications for the reliability of the input obtained, but also excludes the voice of individuals with ID [48].

As part of the Move it, Move ID! project, an intervention was developed in co-creation with adolescents with ID to promote their PA. Although co-creation might lead to more promising interventions, it is often not described in detail and lacks transparency (i.e., what has been done and in what way?). As a consequence, there are few good examples or lessons learned to inspire future researchers. The aims of this study were therefore to highlight elements that must be considered when implementing co-creation and to discuss important lessons learned.

## Methods

### Participants and recruitment

Previous research has learned that recruiting people with ID for research purposes can be challenging. There is less reliance on, for example, advertising or social media to recruit respondents to participate [49]. It may be more important to use an active and personal approach, and to connect with people or organisations close to them: parents, other family members, teachers, health care providers, etc. [49]. For this reason, purposive sampling was used to recruit participants through special needs schools in Flanders, Belgium. In February and March 2021, two physical education (PE) teachers of different special needs schools were contacted. Teachers were asked whether they were interested in being involved with one of their classes in co-creating a PA promotion intervention. Each teacher subsequently suggested one class group to take part in co-creating the intervention. Recruiting in this setting may have several advantages. First, participants know each other, which may facilitate discussion later on during the co-creation process [30, 48]. Second, when the researcher meets the adolescents in their school environment, they or their parents do not have to make the effort to reach a venue (i.e., rely on others to get to a location, transport costs, etc.). Third, the environment is familiar, which can make them feel more at ease. And fourth, in Flanders, children and young people with a disability are classified in different types of special education on the basis of their diagnosis. Approaching adolescents with mild to moderate ID according to the Flemish school system of special education could therefore be explained methodically, instead of testing them or specifically asking for diagnoses.

The participants consisted of two different class groups (group A and B). All adolescents in both class groups agreed to participate. In co-creative literature for the general population, a recommendation of 10–12 co-creators per group is advised, which may also account for dropouts due to the process being conducted over a series of meetings [32, 50]. This guideline was followed as there are currently no guidelines for the specific target group available and the recommendation seemed feasible for this target group as well. Group A comprised 14 adolescents between 17–22 years with a level of mild to moderate ID (M_age_ = 20.33 ± 1.94, 21.4% girls). Four adolescents had a comorbidity of autism. Group B consisted of nine adolescents between 14–15 years with mild ID (M_age_ = 14.22 ± 0.44, 66.7% girls). One adolescent had a comorbidity of autism and one adolescent had a comorbidity of attention deficit and hyperactivity disorder. In total, three (of the 23) participants were part of a sports club. No participants dropped out of the study, but not all participants attended every co-creation session (e.g., because they were sick or suspended).

### Procedure

During the period April-June 2021, six sessions were organised in each class group: one introductory session and five co-creation sessions following the Behaviour Change Wheel-framework. The Behaviour Change Wheel is a theoretical framework to ensure a scientific and systematic development of an intervention [51], making this development process a combination between applying a theoretical framework and co-creation. The application of the Behaviour Change Wheel and the findings that emerged in the process are discussed in more detail elsewhere (see Maenhout et al., *under review*). The co-creation sessions took place at school (in the classroom) during two consecutive class hours (i.e., total of 12 h per group). During each session, the PE teacher was present, as were the principal investigator (PI) (LM, PhD) and one or two assistants of the PI (e.g., master students in Health Promotion, intern, colleague). The PI was the facilitator during all sessions, the assistants were co-facilitators. It was the intention to bring the same co-facilitators in all sessions for the sake of structure and recognisability for the target group, but due to practical circumstances (i.e., exams for the students) this was not feasible. The PI has a master’s degree in Special Needs Education and Disability Studies, with training on how to interact with the target group. She has years of experience as a supervisor at summer camps for children/young people with disabilities and previous working experience as an educator with deaf and hearing-impaired adolescents which provided expertise on easy read language and visualisation. Moreover, the PI is currently based at the Department of Movement and Sports Sciences (Ghent University), and more specifically the unit 'Physical Activity and Health' which has a wealth of experience in developing PA interventions for different target groups. Each co-creation session started with a repetition of the purpose of the project, followed by clearly communicating the objectives that would be addressed in each session, helping to place the present meeting within the overall context of the process [32, 50]. Therefore, a visual schedule was created that recurred at the start of each session, although this had to be adaptable depending on how the process evolved [32]. Furthermore, five ground rules of participation were agreed upon by all attendees, which were repeated during every session: 1) everyone gets a chance to speak, 2) we listen to each other, 3) everyone has a different idea and that is okay, 4) we do not laugh with each other and 5) there are no right or wrong answers [48, 50, 52]. Finally, after each session, both the adolescents as well as the researchers completed a process evaluation form based on the tool developed by Dewaele et al. (2018) for the general population [50] (e.g., satisfaction with participation, feeling at the session, atmosphere, respectful interactions, etc.) [32]. The process evaluation forms for the adolescents were adapted in an easy language. Adolescents marked thumbs (not good—neutral—good) for each statement (see Additional file 1, in the spirit of transparency, the forms were translated in English). They were allowed to do this anonymously to encourage honest answers. The main reason to include these process evaluations was to ensure that the co-creation sessions always took place in a positive, respectful and productive atmosphere by learning how the sessions could possibly be handled differently. When an issue kept cropping up from the process evaluation forms and affected multiple participants (e.g., thumbs down on ‘I understood everything that was said’), the PI tried to adjust subsequent sessions to ensure positive progress. At the end of the co-creation process, all participants, including teachers, received two cinema tickets as an incentive about which they were informed when they decided to participate.

### Ethical considerations and barriers experienced

The participants of the current study were minors and had ID. Consequently, signed informed consents were required from both the adolescents and their parents/legal guardians to allow processing personal data on a legal basis (i.e., GDPR). Unfortunately, receiving signed informed consents from the parents was difficult, as many of them were socially disadvantaged, and lacked the skills and attitude to sign and return the consent form [45, 53]. Moreover, the Covid-19 measures prevented us from actively reaching the parents of the adolescents to verbally explain the purpose and design of the study. To avoid dropout before the start of the study, and make sure that also the most vulnerable adolescents were represented during the developmental process, an alternative strategy was discussed with the Data Protection Officer of Ghent University. Based on an argumentation of the PI, the legal basis (not to be confused with the ethical context), was changed from 'active informed consent' of the parents to one of 'public interest' (i.e., the development of an intervention to improve the physical health and quality of life of this target group fulfils a goal of public interest). Importantly, the use of the ‘public interest’ legal basis did not relieve the researchers of the duty to inform the participants. The participating minors and their parents or legal guardians received accessible and comprehensive information on the design and purpose of the study, as well as on the processing of their data (see consent form of participants with ID in Additional file 2, translated in English). For ethical reasons, active and passive informed consent was obtained from the participants and their parents or legal guardians respectively. The initial introductory session with the adolescents also involved an extensive step-by-step review of the information and consent process with time to discuss, in line with previous studies in the target group [54, 55]. A number of questions were asked to ensure that the participants understood what was being asked of them (e.g., Can you tell me what this research is about? Who decides if you want to participate? What do you do if you no longer want to participate?). The researcher was careful to communicate clearly, using appropriate language for the level of ID and was patient and empathic in her interactions with the adolescents [55, 56]. Going through multiple co-creation sessions with the adolescents also meant that the frequent contact throughout the process allowed the researcher to continuously evaluate whether there was still consent. Adolescents with ID took part voluntarily and could always decide to cancel their attendance.

### Goals and working methods used during the sessions

The facilitators provided working methods adapted to the wide range of knowledge, skills and abilities people with ID have to express themselves [30, 39]. In particular, concise visual materials and interaction were used as both seem to be valued by people with ID [30, 52]. Since no information is yet available in literature on working methods for this specific target group, two sources served as inspiration for preparing the co-creation sessions. Firstly, the Department of Special Needs Education and Disability Studies of Ghent University was contacted to exchange experiences regarding good practices of participatory or co-creative research with participants with ID. They suggested 'Plan P', which is based on the same methodology as co-design, but explained in an accessible way so that people with ID can start their own co-design process [37]. Plan P was developed by 'Onze Nieuwe Toekomst' (‘Our New Future’), a Flemish organisation by and for people with disabilities. Secondly, the online database of De Ambrassade, an expertise centre for everything to do with youth work, youth information and youth policy in Flanders, was consulted. Within this database, different working methods for youth are explained (in Dutch).

The goals of each session and the co-creative methods used to reach those goals are described in Table 1. Pictures visualising methods and materials mentioned in the table can be found in Additional files 3, 4 and 5.

**Table 1:** Description of the goals and working methods used in each co-creation session

Session	Goal(s)	Description of session (e.g., working methods)	Material	Examples
1	1) Introduction of the project and the researcher 2) Going through the informed consent process together 3) Explaining definition of PA, and exploring adolescents’ opinions towards PA	1) Explanation of the project and ground rules of participation 2) **Group discussion** in which adolescents introduced themselves 3) **Statements** regarding PA where adolescents were asked to go to the left or the right side of the room depending on their agreement with the specific statements	1) PowerPoint 2) Informed consents and information letters 3) List of statements the researcher could read out loud	Example statements: “I think exercise is important”, “I think I exercise enough”, “I have enough time to exercise”, “I have enough opportunities to exercise”, etc
2	1) Identify what adolescents (dis)like about types of PA, and what they already do in terms of PA 2) Understanding the facilitators and barriers of PA for adolescents with ID	1) **Write down or draw** all things that came to mind hearing the central theme 'movement and sports' 2) Choose **activity cards** that appealed or did not appeal to them. The chosen cards were then discussed 3) Making **posters** for facilitators and barriers to PA. If this did not come spontaneously, examples were given from the literature using **visual cards** they could pick if they felt that this applied to them	1) Large paper in the centre of the room with several markers 2) Activity cards which contained images of different sports or physical activities 3) Red and green poster 4) Visual cards of facilitators and barriers to PA	Example questions using activity cards: Why do you (dis)like (the activity)? Did you already do (the activity)? Can you tell me about the last time you did (the activity)? Do you know where to get information about (the activity)? Who did you do (the activity) with? Where did you do (the activity)? Who chose to do this activity? Where do you do (the activity)? Example questions using posters: What makes it hard for you to do sports/to do PA? What makes it easy for you to do sports/PA?
3	Explore the most important intervention goals on which the intervention should focus	1) Previously mentioned barriers were presented and explained using a **micro-meso-macro model** 2) Possible intervention goals were visualised on a **poster**. During explanation, adolescents could **vote** with a green/red sticker whether they thought this goal should be included in the intervention or could be left out 3) Adolescents worked together to **rank** the intervention goals from most important to least important	1) Micro-meso-macro model of the identified barriers in session 2 2) Posters on which the 16 possible intervention goals (based on barriers mentioned in session 2) were visually represented 3) Green and red stickers to vote	Example of an intervention goal: from 'not knowing what there is about exercise', 'not knowing where I can exercise', 'I need information', 'not knowing what suits me', 'not knowing how much I should exercise', 1 poster was made with the intervention goal 'knowledge provision'
4	Identify how to implement behaviour change techniques (BCTs) to be accessible and engaging for adolescents with ID	For each selected intervention goal (session 3) a **story** about a fictitious adolescent was described, so that the participants could empathise with the story. Next, adolescents were asked in which way they thought the person would feel most helped. First, they were asked to brainstorm on this themselves in **small groups.** Second, concrete examples were given from previous interventions or apps on a **PowerPoint slide** for the adolescents to **vote** on with red or green cards	1) PowerPoint slides with picture of fictitious adolescent and examples from other interventions or existing apps 2) Green and red cards	Storytelling: ‘This is Marie [picture]. Marie is 17 years old. Marie does not like to exercise. Marie has already noticed that it is easier for her to exercise when she is encouraged to do so or when movement is part of a game. How could we encourage Marie to exercise?’ BCT ‘reward’, examples: Getting coins to dress up an avatar; receive a badge; social rewards through likes; material rewards like a power bank, water bottle, voucher for a (sports) shop; earn coins online so that they can exchange them for a discount at a shop, etc
5	1) Explore which apps adolescents are currently using 2) Identify facilitators and barriers to app use 3) Identify design preferences	1) **Group discussion** in which it was asked which apps adolescents mostly use in their daily lives, and what they liked about these apps 2) **Smaller groups** received an **iPad** with some **apps** they could **test**. Each group was asked to write down on a green sheet what they liked about the app(s) and on a red sheet what they did not like. Afterwards, the findings were shared with the larger group	1) iPads and downloaded apps 2) Green and red piece of paper	Example questions of group discussion: Which apps do you use? What do you (dis)like about these apps? What makes those apps easy (or difficult) for you to use? What do you (dis)like about the design? When do you find apps not interesting/too difficult? Do you know/use apps that have something to do with PA? Examples of apps that were tested: Fitbit, #LIFEGOALS, SideKick Health, Seven, Zombies Run, etc. Apps were selected on the basis of familiarity with young people (e.g. Fitbit, Zombies Run), previously developed apps within the research group (i.e., #LIFEGOALS [57]) and other apps developed for the target group within research (i.e., SideKick Health [58])
6	1) Explore the opinion of adolescents with ID on a possible intervention idea 2) Find out which incentives young people with ID prefer 3) Probe how adolescents feel about the use of an accelerometer during an intervention	1) Adolescents were divided into **smaller groups** to reflect on an intervention idea presented by the PI, supervised by a member of the research team 2) Using a **PowerPoint slide**, examples of possible incentives were presented whereby adolescents could indicate their preference as to which incentive they liked best 3) After a general explanation what an accelerometer is, adolescents could **test** some **accelerometers** themselves (wrist/waist worn) and give their opinion	1) PowerPoint slide with intervention idea 2) PowerPoint slide with examples of incentives 3) Accelerometers adolescents could test (an Axivity AX3 (with wristband) and an Actigraph GT3X + (with waist belt))	Example questions on the intervention idea: What is the best way to reach young people and ask them to participate? How to communicate all information about the project? Example questions probing the accelerometers: What do you think about an accelerometer? Would you mind wearing it? Which wearing location would you prefer? What do you think about the length of time you have to wear it? How can we remind you to wear it? Would you keep a diary? Can parents help?

### Measurement instrument

The reflection forms that the researchers (both PI and co-facilitators) completed after each session, formed the basis of this paper. The aim of this form according to the researchers that have developed it is to feed discussion about the co-creation process, and not to be used as mere quantitative measure [50]. The reflection form started with 10 open questions (e.g., “what went well”, “how can we bring out the qualities in the group”, etc.), followed by a table with 19 statements where the degree of agreement could be indicated on a 5-point Likert scale (see Additional file 6). The goal of this reflection form was to stimulate the researchers to reflect as broadly as possible, whereby both positive and working points could be highlighted (i.e., what went well? And what c/should be improved?). In addition, the researchers were free to supplement reflections that were not specifically asked for in the form. Furthermore, any extra information the PE teacher provided before, after or during breaks of the sessions was written down in the reflection forms of the PI. In the reflection forms, the PI also reflected on the results that came from the adolescents' process evaluations after each session. In this way, the adolescents' process evaluations were also included in this paper. Additional file 7 shows a description of the results of those process evaluations per session and per group. The process evaluation forms of the participants with ID were indirectly included in the data analysis through the reflections on them by the PI, but were not directly included in the analysis process.

### Analysis

Researchers’ reflections resulted in a total of 29 forms. Inductive thematic analysis was applied to map the most important results. Thematic analysis is a method of identifying, analysing and reporting on themes and sub-themes within data [59]. Inductive or “bottom-up” thematic analysis codes the data without a pre-existing framework [59]. To code the data, we followed the analysis process described by Braun and Clarke (2006), who divide the process into six separate phases [59].

The first step was to familiarize with the data (i.e., reading through forms several times). As transcription of the material was not needed, the PI (LM) read the reflections and wrote some general findings down. In the second step, the data were read again, and initial codes were generated by two independent coders (LM and SC) using qualitative data analysis software NVivo 12.0. A second researcher (SC)—who was not present during the co-creation sessions—analysed the data separately to keep the analysis of the data as objective as possible. A consensual approach was adopted, in which inconsistencies were discussed between the two analysing researchers. In the third step, codes were brought together in different themes by LM, establishing a first differentiation between main and subthemes. Next, all data was read again but with the identified themes in mind. This was done to check whether the data was well captured by the themes. In the next steps, the researchers LM and SC discussed and defined the themes to finally reach a fully analytical narrative with vivid quotations. Lastly, the results were reviewed by the assistants involved in the co-creation sessions to check whether the identified themes matched their experiences.

The COREQ (COnsolidated criteria for REporting Qualitative research) checklist [60] and GRIPP2 (Guidance for Reporting Involvement of Patients and the Public) reporting checklist [61] were consulted to ensure that the data was reported as broadly as possible and were added in Additional files 8 and 9 respectively.

## Results

**Fig. 1:**
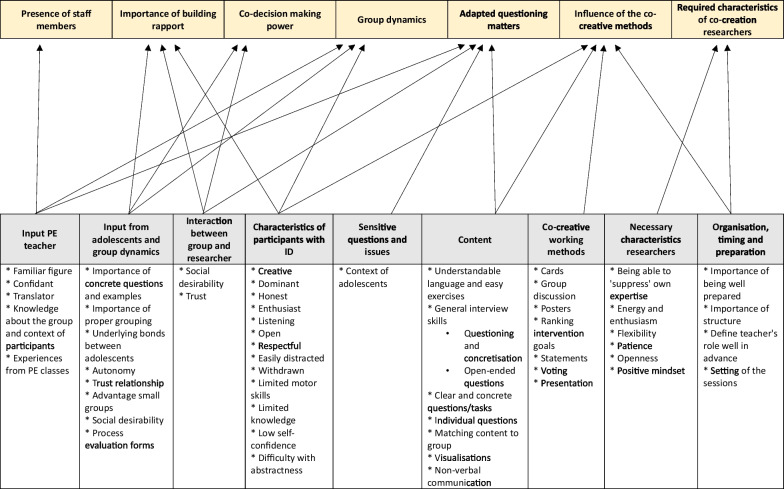
Overview of the main themes and codes that contributed to those themes

Figure 1 provides an overview of the seven main themes that emerged, and which codes contributed to that theme (i.e., what a reader can expect to find in terms of information within a theme).

### Presence of staff members

The presence and participation of the PE teacher during the sessions was felt to be of added value for several reasons. First, a number of adolescents saw the teacher as a familiar figure and confidant, which made them more inclined to participate. For example, it was noticed that adolescents first whispered an answer to the teacher before mentioning it in front of the whole group, probably to be sure their answer was meaningful. Second, the teachers acted as translators if the question was not entirely clear to the adolescent. Third, they knew the context of the adolescent better than the PI did. Consequently, either questions asked by the researcher or answers given by the adolescents could be contextualised by the teacher so both sides understood each other sufficiently. Moreover, tips could be given to the PI towards adapting questioning.

*“Within co-creation, the teacher also participates: writes something down now and then (e.g. on a large sheet), participates in discussions, ... You notice that pupils are at ease as a result. The teacher's input can also help to put things in context: e.g. what they mainly do at school, why they do not like certain things (e.g. swimming).”* (PI, session 2, group A)

Fourth, PE teachers were able to give input from their experiences in physical education lessons. This helped because the teachers thought about the project from a different view, providing a fresh perspective to the topic.

*“The presence of [teacher name] during the sessions is also a great asset to this group. [Teacher name] first lets the group answer by itself, but now and then he completes the questions or challenges the group to think 'differently'. E.g.: if everyone puts a green sticker on an intervention goal, he can give an example of a red sticker, which makes the group reflect on it.”* (PI, session 3, group A)

Moreover, the teachers were able to contribute to the structure of the sessions, as they knew the group better. For example, each session was planned to begin with a reiteration of the project's purpose, where the session was specifically situated within the process and what the agreements were within a session (see ‘Procedure’). In consultation with one of the two teachers after the fourth session, it was decided not to repeat this anymore the other two remaining sessions because she noticed adolescents quickly lost their focus because of it. The teacher suggested it would be better to jump right in and get them engaged immediately.

### Importance of building rapport

The first session was a real search for the researcher(s): not knowing the group, not knowing the teacher, not knowing the context adolescents live in, etc. Throughout the sessions, the researcher got to know both the group and the teacher better and gradually started to build rapport with the participants. After a few sessions, it was for example noticeable that during breaks, conversations were held that were often outside the scope of the project. Building that bond is necessary to get as close as possible to the perception of the adolescents and to give them the confidence to share their opinion. Throughout several sessions, the researchers could observe that the participants felt increasingly at ease, for example by sharing their openness about more difficult topics such as their home situations.

*“By creating more trust, the participants were more open about their experiences and feelings. This is something that has to grow throughout the process.”* (masters student, session 2, group B)

Moreover, a relationship of trust not only seemed important between the participants and the researcher, but also among participants themselves.

*“They trusted each other and everyone could speak their minds. They knew a lot about each other and picked up on it.”* (masters student, session 2, group A)

### Co-decision making power

An unprecedented step for the researcher to take was daring to let go of control, and give the co-creators the autonomy and ownership to steer the process. If the PI was in doubt or lost as to which methods worked best for the group, or how best to address the participants, the participants themselves were asked how they thought they could best be approached. Moreover, by means of the process evaluation forms that the adolescents completed, the researchers were able to check how the participants had actually felt during the different sessions, so that appropriate adjustments could be made. These forms gave the participants a voice to guide the course of events.

*“The main conclusion is that the majority of the adolescents did not understand everything that was being said. In the next sessions, I will have to pay attention to speaking more slowly, more clearly, more concretely.”* (PI, session 1, group B)

*“Very positive process evaluation. Almost all students gave a green thumbs up to all statements. The arrangement of three separate groups with three facilitators, without any feedback to the whole class, seemed to suit this group best.”* (PI, session 6, group B)

### Group dynamics

The co-creation sessions took place in two different groups, whereby it was immediately apparent that both groups were different and consequently required a different approach, even though the sessions for both were initially set up in a similar way.

In both groups, it was striking that the more articulate individuals quickly took the lead, and the more silent adolescents often just followed them. However, both groups were different in that aspect. Adolescents from the older group (17-22y) seemed to be stronger in having their own opinions and expressing them, even if these did not entirely correspond to the opinions of the more expressive participants or the majority. The youngest group (14-15y) seemed to struggle more with this. Most of them had a withdrawn attitude. It could be inferred that there was a lot of compliance in the input they provided; not compliance towards the researcher(s), but towards their more outspoken peers leading to little interaction between them. The teacher of the youngest group explained that this class group was actually a combination of three different very small class groups, who did not always attend lessons together. The oldest group, on the other hand, had been in the same class for years and seemed to respect each other's opinions despite their individual differences. The different group dynamics that prevailed are closely related to the previous paragraph that indicates that there should be an atmosphere of trust among the participants in order to make co-creation a success.

*“In the exercise where they had to work together [ranking the intervention goals], there was very little cooperation, but rather everyone working individually. The opinion of the three outspoken girls was followed the most. I don't know if the rest of the group agreed with everything, as there was no discussion. When I asked if everyone agreed or if someone would put an intervention goal in a different place, there was again no response.”* (PI, session 3, group B)

To counteract these groups dynamics, the decision was made to divide the youngest group into their usual smaller class groups as much as possible during the course of the sessions. This way, everyone would be motivated to speak up and express their opinions honestly.

*“There was good interaction and the students cooperated well. The students dared to speak their mind honestly. This went better because of the smaller groups.”* (master student, session 2, group B)

*“In the small groups, they feel at ease and dare to talk more. When this has to be shared with the larger group, it became quiet again.”* (PI, session 4, group B)

### Adapted questioning matters

How questions were asked proved to have an impact in this target group. First, it was important to present the questions as concretely as possible, with many (visual) examples for clarification. We made a lot of effort to use language appropriate to the target group and to use other, more easy words (i.e., no jargon) to explain concepts if it was felt that the participants did not understand everything (e.g. when there was no response to questions). However, it seemed difficult to assess what participants did or did not understand. For example, due to the division in smaller groups, some participants dared to tell the researcher for the first time that they did not understand while they did not do this, or hardly at all, in (earlier) group discussions.

*“Words that I have used several times over the course of the sessions are actually unclear to the adolescents. For example, the word 'motivate'. [Student name] did not understand what I actually meant by this, even though I think I used this word in every previous session. Somehow, this made me a little insecure about the previous sessions, because I have the idea that they did not understand everything I asked them then either.”* (PI, session 5, group B)

Therefore, during breaks, the PE teacher was consulted whether the questions were clear enough for the group. Participants often really needed a choice between two options (i.e., ‘do you prefer this or do you prefer that?’). It was therefore experienced difficult for the researcher to find a balance between asking a concrete question and not wanting to fill in too much for the adolescents. Furthermore, adolescents who were more in the background were more encouraged to take part in the conversation when questions were asked more individually.

*“The whole class took part in the group discussion. If the somewhat quieter participants were asked a question directly, they also spoke openly about their experiences.”* (master student, session 1, group A)

It was also important to keep the adolescents’ context in mind when asking questions and the sensitivity in language use in that respect. For example, many participants came from a more socially disadvantaged home situation, where not both parents were present. When asking a question, it was then important to talk about 'parents' or ‘at home’, rather than 'mum' or 'dad' specifically, as this could hurt the feelings of young people where 'mum' or 'dad' was not present, leading to resistance in the co-creation sessions.

### Influence of the co-creative methods

Adolescents in both groups were hesitant when being asked a general question in front of the whole group, for example *'which apps do you use and what do you like/dislike about them'*. With such a general question, it usually remained silent in which a wait-and-see attitude was adopted. It was remarkable that the adolescents became more relaxed when offered a very concrete exercise (e.g., statements or test apps on an iPad) and visual materials they could interact with (e.g., cards with pictures or posters they could vote on). Getting creative was an entry point for further discussion: in the statement exercise, adolescents could indicate why they were standing on one side of the room and not on the other side; using the activity cards, they could explain why they had chosen a particular card, or why they had voted for a particular poster; when testing different apps on the iPads, adolescents could give their input on that basis.

*“[Teacher name] indicated that using the [physical activity] cards was a good method for the adolescents, that she was stunned by some of the young people's cooperation and that she will certainly use this way of working in her lessons in the future.”* (PI, session 2, group B)

Moreover, creative/interactive methods were also a way of getting less talkative adolescents to integrate their opinions as well. For example, in the poster exercise where adolescents had to vote on intervention goals, verbally expressing an opinion was difficult for some, but through the sticker-method they could at least indicate whether they agreed the intervention goal should be integrated (green) or not (red).

Some co-creative methods were more successful than others. The session with the visual cards showing examples of barriers and facilitators to PA provided considerable input due to the comprehensibility for the participants. Both teachers were enthusiastic about this method and indicated that it was also interesting for them to discover which methods did or did not work well with their students. One teacher indicated that this was a very good way to better understand young people with ID’s thoughts concerning movement.

*“Splitting the group in two and making the assignment very visual (with cards: both different types of physical activities and barriers/facilitators to physical activity) really helped the adolescents. In both exercises, the groups participated well and a lot of interesting information came out.”* (PI, session 2)

In the session on intervention goals, goals were displayed visually per poster, and adolescents could vote with stickers. This session proceeded smoothly because of the interaction and active participation of the adolescents. When placing a sticker, some adolescents already explained more about why they placed a certain sticker without even being asked. Ranking the intervention goals, however, seemed to be difficult for both groups, but for a different reason. In the youngest group, there was no cooperation: more outspoken participants worked individually, and the others watched. The oldest group could not make a choice which goal they considered more important, and lumped everything together. Only two of the 16 goals were clearly chosen as less relevant. It could have worked better if the larger group was divided into smaller groups for this exercise, increasing discussion.

Getting to test apps on an iPad was a success. The adolescents were curious about the apps, so they often enthusiastically tried out different things. By allowing adolescents to test apps and think aloud, it was possible to learn first-hand what they found important.

A technique that worked less well was the story-telling during the fourth session. Whereas the youngest group was involved in the stories: *“do you really know this person, where does he live?”*, the oldest group did not react to this. However, it became apparent that this method was flawed due to the abstract nature of the task. Adolescents were asked to think about something that was not there yet, or without knowing what it might look like, leading to the fact that they did not really understand what the exercise was about or what was expected of them. In contrast, the previous sessions included concrete exercises, with clear direction, and adolescents were much more interactive. Due to the struggle with this abstractness, it was decided to skip the BCTs that did not have specific examples (e.g., valued self-identity, identity associated with changed behaviour), as it was noted that there was little or no response. In the last session, we did also not proceed with asking adolescents to think about a possible intervention on their own in small groups, but instead for the researcher to present an intervention idea that was based on the input adolescents provided in previous sessions and ask for their feedback.

*“This session is difficult for the adolescents because they have little idea of what an app can do to meet their chosen intervention goals.”* (teacher, session 4, group B)

*“The process evaluation forms mainly showed that the majority of adolescents did not understand everything that was being said. A possible explanation could be that these two rather theoretical sessions (3 and 4) are rather abstract, and that it is difficult for young people to imagine an app that does not actually exist yet.”* (PI, session 4, group B)

Furthermore, showing examples on a PowerPoint slide seemed to hinder the imagination of the participants. Using this method, participants were already being pushed in a direction somehow. This became clear with a PowerPoint slide showing possible incentives. Participants gave their opinion on incentives that were presented, but no other input, apart from the slide, was given.

Tasks where participants had to write down certain things also hampered the process. A method in which they can work creatively or interactively suited them better.

*“Part of the exercise was for the groups to write down what they had come up with. It struck me that writing this down was already a barrier in itself. The young people preferred to remember what they wanted to say, rather than writing it down.”* (PI, session 4, group A)

Lastly, the setting in which the co-creation took place could have played a role as well. For example, during the second session in the oldest group, the weather was very nice, so it was decided to go outside with a group, which positively influenced the discussion dynamics.

### Required characteristics of co-creation researchers

By conducting these sessions, it was noted that being a co-creative researcher requires enthusiasm, patience, flexibility and openness. First of all, it was important to remain enthusiastic and generate interest among participants by organising the sessions in an exciting way. It was noted that enthusiasm ignites: by being enthusiastic in front of the group, the group in turn reacted engaging and enthusiastically as well. Moreover, patience was needed. If the group did not understand the researcher or question, it was key to keep searching for ways where a similar language could be found that maximised cooperation, for example by using creativity. This required energy, but there was no point in giving up if something did not go as expected. However, when the co-creative process did not go smoothly, efforts were made to adopt a positive attitude: colleagues reminded the PI that no answer is also an answer. It was perceived helpful to share uncertainties with the research team during the process. Furthermore, despite a good preparation and a clear structure at the beginning of a session, a lot of flexibility was needed from the PI. Usually, a session did not go as planned at all. For example, teachers had to reschedule sessions for various reasons (e.g., excursions, info days at school, teachers were not present themselves due to private reasons, etc.), or there was not enough time to discuss everything that was planned, or vice versa there was too much time left without having prepared another exercise.

*“The statements ran out pretty quickly. I asked a number of other questions, but felt less prepared.”* (PI, session 1, group B)

Moreover, it was noticed that certain methods did not work for one group, whereas they worked for the other group, or that it was just not working for both groups. A session had to be prepared on fairly short notice (i.e., one week) based on the input from the previous session(s). In the context of flexibility, it was important to have a back-up plan ready if it was felt that something was not working, or if there were time constraints. Having a back-up plan ready, made researchers feel more confident.

*“Co-creation is also about letting go of control and initial plans. It is searching for what works best for the group you have in front of you.”* (PI, session 4, group B)

Lastly, it was important to be open to what interests the participants. This required adaptability to letting go of what the PI had in mind based on the literature. Moreover, in the context of openness, the PI also shared something about herself from time to time. This helped to build rapport with the participants, as it was noted that participants were more open and vulnerable when the researcher did the same. In this respect, a balance had to be found for the researcher in how far things of her personal life were shared.

## Discussion

### Principal findings, practical implications and future recommendations

This paper reflects on the co-creation process during the development of the Move it, Move ID!-intervention from the researchers' perspective. Butler et al. (2012) already stated a decade ago: ‘‘*we would really like other researchers to write about their experiences about doing research together, both the good and the bad, so we can learn from each other*’’ ([62], p. 142). Notwithstanding this call for transparency, we remain often in the dark on this topic when reading papers. To address this issue, we described the co-creative process used to develop a PA intervention for adolescents with ID in detail and we formulated seven elements that could be taken into account to make co-creation feasible in this target group, being 1) the presence of staff members, 2) building rapport, 3) co-decision making power, 4) the adaptation to the group dynamics at play, 5) the necessity of adapted questioning, 6) the influence of the co-creative methods and 7) the required characteristics of a co-creative researcher. In what follows, the most striking findings are discussed in more detail. Although the focus of this research team was on developing a PA promotion intervention, we assume that the results regarding lessons learned would not have differed greatly had a different behaviour been central.

The presence of staff members (here the PE teacher) during the co-creation process was seen as an added value for several reasons. Informal talks with the PE teacher(s) during breaks or after the sessions were seen as beneficial to gain a deeper understanding of the health problem being addressed [32]. However, during the sessions it became clear that the teachers were not well informed about their role. In preparing the co-creation sessions, the PI mainly focused on finding ways to involve the target group in the discussions, whereby the role of the teacher was somewhat lost sight of. This only emerged when the teacher asked questions or spontaneously took on this role. Teachers did not know whether they were allowed to participate. It is therefore considered important to clearly communicate their role before the sessions start. Of course, the presence of a teacher could also have a downside: e.g. young people who would prefer not to say certain things because their teacher is present. This was however not noticed within the two groups of this study. Both classes had a good relationship with their teacher. To decide whether to include the teacher in the co-creation process, a pre-observation of the group dynamics could provide answers to this question.

Within this co-creative process, co-decision-making was pursued by having the participants give their opinion on the process through the process evaluation forms after each session, as well as by asking for alternative approaches if a session did not run smoothly. The participants were co-creators, which means that they were on the same level as the researcher. The aim was to strive for an equal relationship, where the researcher’s opinion had no more weight than opinion of the others. A subsequent session was prepared fully based on the input received from the previous session(s). However, the content and flow of the sessions was guided by a theoretical framework, the Behaviour Change Wheel (see Maenhout et al., *under review*). This guidance was chosen to support the process theoretically, as well as to provide clarification within a project application. However, while trying to follow a guide, a difficult balancing act was noted with full co-decision-making power of participants. Co-creative research often takes the researcher to places that were not expected, in which co-decision-making is established by following the path participants are taking. Nevertheless, researchers cannot free themselves of their theoretical and epistemological commitments [59]. Co-creative research cannot be fitted into pre-defined categories or themes, but data can also not be coded in a vacuum. The use of the reflection forms taught us that it is important to reflect sufficiently as a researcher and to exchange thoughts and findings with, for example, the team or colleagues so as to remain aware that the researcher’s view is only one view of reality. Abma et al. (2019) expressed this perfectly by stating that *"it is not just about exploring others' frames but also critically reflecting on our own as we deepen the inquiry process"* [63]. A recommendation for future research could be to add a person/persons from the target group to the research team (i.e., co-researcher(s)) already at the start of the project to boost co-decision-making. According to Smith et al. (2022) this is called *equitable and experientially-informed research* in which people with relevant lived experience/experiential knowledge are seen as essential to the research [42]. Equitable partnerships between the different contributors are promoted and maintained throughout the entire research process, so a variety of perspectives are included and not just that of the (principal) researcher(s). Two studies could provide inspiration concerning inclusive research with people with ID: 1) a study describing the role, barriers and supports of co-researchers with ID in Chile [64] and 2) a consensus statement on how to conduct inclusive health research with people with ID [47].

When preparing the co-creation process, it was remarkable that there are currently few recommendations on how best to approach co-creation research with this target group. There are studies that make general recommendations but lack practical examples to get started [42, 47, 54]. For example, for the specific working methods, the PI had to fall back on previous experiences she had with the target group, as no information is available in literature on which concrete methods suit our specific population, context and goals best. This study showed that conducting co-creative research with this target group is feasible when adjustments are applied. Adjustments appear to be particularly important in terms of communication (i.e., the necessity of adapting questioning): (young) people with ID have differences in language use; difficulties with abstract thinking and recall; are inclined to give compliant answers; experience difficulties with written information; and are overall an extremely heterogeneous group in terms of communication capabilities [41, 45, 54]. It is therefore important to focus on concrete experiences/tasks, read exercises out loud, provide accessible language and illustrations and even develop communication tools in collaboration with them [47, 54]. In this respect, the use of creative working methods to initiate conversation was extremely valuable [65]. The reflections of the researchers showed which methods worked better than others. For example, a concrete exercise with visual cards worked considerably better than an abstract exercise in which a story about a fictitious adolescent was told. Although these examples of methods have now been applied to this specific target group, they could potentially also be used within the general population to promote creativity and maximise interaction. Currently a lack of participation is often seen as synonymous with apathy, but maybe it can rather be seen as a need to find entry in other ways. It would therefore be of great value for future co-creative researchers to be able to consult a taxonomy of creative methods that can be used. As an example, the book ‘*Seldom Heard Voices: The how and why of meaningful collaboration*’ (2022) [45] was published after carrying out this co-creative process. This is the first book that shares experiences and examples of service user involvement with communities of seldom heard voices, including a chapter which described facilitation tools to support engagement with people with ID. These facilitation tools can be considered the same as the described working methods in this paper. Each tool has a description and sums up limitations or considerations. Together with the methods described in this paper, this can provide a first starting point for such a taxonomy. Creating a taxonomy of working methods is also one of the objectives of the Health Cascade Project. Health Cascade is a Marie Skłodowska-Curie Innovative Training Network funded by the European Union’s Horizon 2020 research and innovation programme aimed at delivering the rigorous scientific methodology to secure co-creation as an effective tool to fight public health problems [66]. It would be interesting if the Health Cascade Project could also focus on specific target groups (e.g., which methods would be recommended in which target group), and therefore would not only make recommendations for the general population, since co-creation is as vital with those vulnerable target groups. In this way, different disciplines can learn from and strengthen each other within co-creation.

The use of various creative working methods required the researcher to be well prepared on the one hand, but also to be flexible and make decisions in the moment on the other hand. How well the PI might have prepared a protocol about how to conduct co-creation, the protocol often had to be abandoned based on the group the researcher had in front of her and the input that was given. This, however, can be difficult because funding bodies often expect a detailed plan before funding for research projects can be provided [42]. This shows the messiness of co-creative research. A possible way of being better secured against this, might be to make an assessment of the group dynamics before conducting co-creation, if possible. For example, when preparing the co-creative research in a class context, it could be an option to first go observe the class group during their usual lessons or to spend more time getting to know each other at the beginning of the process, e.g. first be physically active together and then start with the workshops. Creative methods could subsequently be (better) chosen based on that observation. Some participatory or co-creation studies even describe selecting eligible persons (e.g., persons who had certain prerequisites in mobility, language skills, and ability to concentrate) with the help of people who already know them [41]. The success of co-creation (methods) largely rely on the relationships that exist and emerge. A good working environment in which genuine feelings can be expressed and in which there is a shared understanding is necessary [32, 54]. During this co-creation process, groups only came to discussion when everyone’s input was valued equally (e.g., no formation of hierarchy) [50]. When there was no trust, it was difficult to encourage participants to get involved, leading to no interaction and no flow of ideas and input (i.e., different group dynamics in both groups). In this respect, it is important for researchers to learn how to build relationships of trust (as this is a fragile process in constant need of (re-)negotiation [42]), but also to have the opportunity to collaborate with pre-existing groups or familiar people in which there is a respectful atmosphere [30, 48], which can only be discovered by getting to know the group before the co-creation process starts.

Numerous benefits have been listed in literature in regard to co-creation. For example, co-creation for participants themselves would lead to empowerment; gained skills, confidence and experiences; a sense of contribution and respect; etc. [32, 45, 47, 63]. For researchers, it would in turn lead to the development of more appropriate interventions; the identification of appropriate research methods or creating new methods; the generation of novel and conceptually rich knowledge; the advance of innovative theories and new concepts; and the acquisition of new skills [41, 42, 54]. However, also barriers and challenges have been described [42]. For example, co-creation research has an often lengthy timescale. Thus, it is also important to assess whether co-creation actually leads to more acceptable, effective and sustainable interventions that are also cost-effective. There is still too limited information on the (cost) effectiveness of co-creation, which should be addressed by future research.

### Limitation and strengths

The main limitation of this study is the primary focus on the researchers’ reflection forms as data material. An attempt was made to obtain a reflection from adolescents with ID using the process evaluation forms with thumbs up/down, but no direct reflections or quotes from adolescents or PE teachers are available to include within this manuscript. We recommend future researchers to include reflections from different perspectives on the co-creation process within data collection, as it might provide supplementary information for future (co-creation) researchers. Second, the PI (LM) analysed her own reflection forms to be able to write the results of this study, creating bias. This was countered by also analysing reflection forms of co-facilitators, as well as involving a second researcher (SC) to code the data. It can therefore be concluded that the PI may have analysed more deductively as she witnessed the whole process and already had ideas about themes in her head, and the second investigator more inductively. Third, a wide age range of adolescents with ID was chosen to participate (13–22 years old). It was noted and described that the two groups differed greatly both internally (content) and externally (working methods), raising the question how to prioritize different views. Choosing a tighter age limit (e.g. 13–16 years old or 17–22 years old) seems recommended in future co-creation research. A fourth limitation of the study is that the choice was made to work only with adolescents with mild to moderate ID, which means that the findings cannot be extended to the target group of severe or profound ID. Future research needs to focus on the representation of all people with ID in health research, notably how to actively involve people with severe and profound ID directly or by proxy [54]. Last, next to following education in special need schools, adolescents with ID also have the possibility to follow (inclusive) education in mainstream schools in Flanders, Belgium. Nevertheless, the percentages of students receiving education in special need schools remain high (partly due to a lack of (individual) support in general education). Because of practical reasons, we only recruited adolescents from special secondary education for this study and not adolescents with ID from inclusive education. However, within the effect study of our intervention, we will very specifically recruit adolescents with ID from inclusive education as well. The greatest strength of this research is that it is, to the best of our knowledge, the first study that involved adolescents with ID in intervention development aimed at increasing PA. This fits perfectly with the “Nothing About Us, Without Us”-motto which is echoed in the philosophy and history of the Disability Rights Movement. The motto encapsulates a fundamental shift in perspective towards a principle of participation and integration of persons with disabilities. Co-creation enables people with ID to participate and thus to increase control over and to improve their health. Second, this is the first study to provide concrete tools for implementing co-creation with this target group. Third, by having both the facilitator and co-facilitators reflect after each session, an attempt was made to identify different lenses on reality. From this, important lessons could be formulated that might be relevant for other researchers who want to start a co-creation process.

## Conclusion

This study formulates seven elements that could be taken into account to make co-creation feasible within this target group. In particular, the need for innovative, concrete (non-abstract) and creative working methods that allow for the inclusion of those with different communication styles is emphasized. Transparent descriptions of the different steps taken, working methods used and lessons learned could be a first step to turn co-creative research into an evidence-based methodology.

## Supplementary Information

**Additional file 1**. Process evaluation forms of adolescents with ID.

**Additional file 2**. Informed consent forms of adolescents with ID.

**Additional file 3**. Pictures of co-creation session 2.

**Additional file 4**. Pictures of co-creation session 3.

**Additional file 5**. Pictures of co-creation session 6.

**Additional file 6**. Reflection form of researchers.

**Additional file 7**. Description of the results of the adolescents' process evaluations per session and per group.

**Additional file 8**. COREQ (COnsolidated criteria for REporting Qualitative research) Checklist.

**Additional file 9**. GRIPP2 Reporting Checklist - Long Form.

## Data Availability

All data generated or analysed during this study are available upon request.
