# Supplementary material for: Co-creating an intervention to promote physical activity in adolescents with intellectual disabilities: lessons learned within the Move it, Move ID!-project
Source: Res Involv Engagem. 2023 Mar 19;9:10. doi: 10.1186/s40900-023-00420-x (PMC10024913; doi:10.1186/s40900-023-00420-x)
Supplement: Supplementary file 1 — Additional file 1. Process evaluation forms of adolescents with ID. [file 40900_2023_420_MOESM1_ESM.pdf]

Move it, Move ID!

HOW DID I LIKE THE CLASS DISCUSSION?

Put a cross X by the thumb that suits you best:

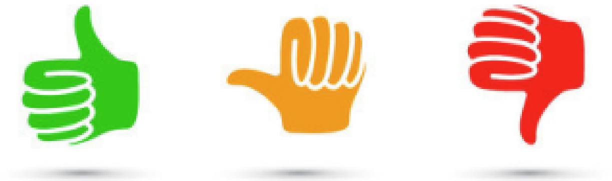

I was able to give my opinion	☐	☐	☐
Others listened to my opinion	☐	☐	☐
I listened to the opinions of others	☐	☐	☐
I dared to say everything I wanted to say	☐	☐	☐

UNIVERSITEIT GENT

fwo

Laura.Maenhout@UGent.be - Watersportlaan 2, 9000 Gent | 09 264 63 21

Move it,
Move ID!

HOW DID I LIKE THE CLASS DISCUSSION?

Put a cross X by the thumb that suits you best:

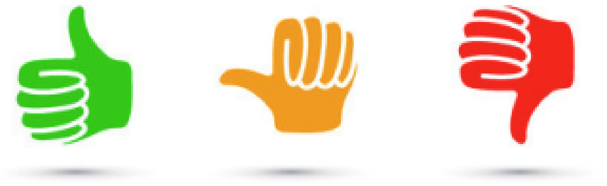

I had a good feeling about the conversation			
I learned something			
I understood everything that was said			
I found it interesting			
I understand what the purpose of this class discussion was			

UNIVERSITEIT GENT

fwo

Laura.Maenhout@UGent.be - Watersportlaan 2, 9000 Gent | 09 264 63 21
