# Supplementary material for: Co-creating an intervention to promote physical activity in adolescents with intellectual disabilities: lessons learned within the Move it, Move ID!-project
Source: Res Involv Engagem. 2023 Mar 19;9:10. doi: 10.1186/s40900-023-00420-x (PMC10024913; doi:10.1186/s40900-023-00420-x)
Supplement: Supplementary file 2 — Additional file 2. Informed consent forms of adolescents with ID. [file 40900_2023_420_MOESM2_ESM.pdf]

MOVE IT, MOVE ID!

Informed consent adolescent (1)

Put a cross X if you agree:

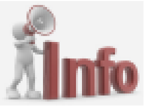

I received information about the research

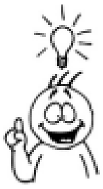

I understand what the research is about

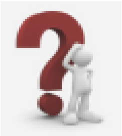

I was able to ask questions if I did not understand something

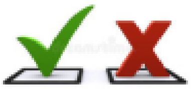

I choose to participate in the study myself

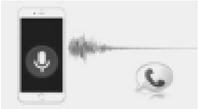

I agree to have my voice recorded during the conversations

Laura.Maenhout@UGent.be - Watersportlaan 2, 9000 Gent | 09 264 63 21

MOVE IT, MOVE ID!

Informed consent adolescent (2)

Put a cross X if you agree:

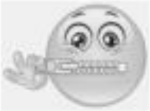

I understand that information about me will be kept private ☐

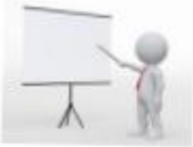

I know researchers will give presentations or write articles about what is told ☐

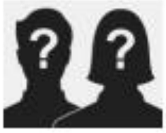

I know no one will know my name ☐

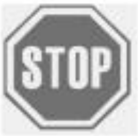

I know I may stop participating in the study at any time ☐

Laura.Maenhout@UGent.be - Watersportlaan 2, 9000 Gent | 09 264 63 21

MOVE IT, MOVE ID!

Informed consent adolescent (3)

Put a cross X if you agree:

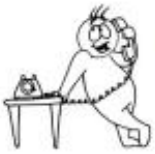

I know I can ask the researcher (Laura) after the interviews how the project is going or how the app looks like ☐

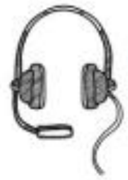

I know other researchers can also listen to the conversations ☐

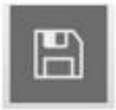

I understand that information about me will be kept securely for 5 years ☐

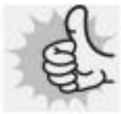

I would like to participate in the study ☐

Name:

Date:

Laura.Maenhout@UGent.be - Watersportlaan 2, 9000 Gent | 09 264 63 21
