# Supplementary figures and images for: Co-creating an intervention to promote physical activity in adolescents with intellectual disabilities: lessons learned within the Move it, Move ID!-project
Source: Res Involv Engagem. 2023 Mar 19;9:10. doi: 10.1186/s40900-023-00420-x (PMC10024913; doi:10.1186/s40900-023-00420-x)

SUPPLEMENTARY FILE 3: CO-CREATION SESSION 2

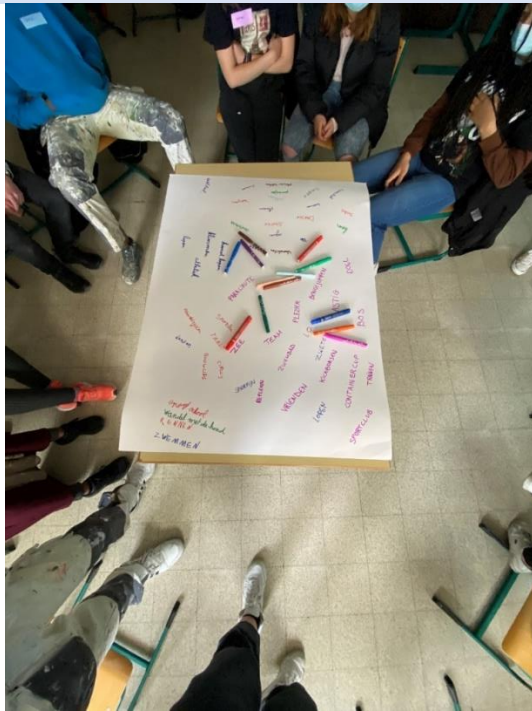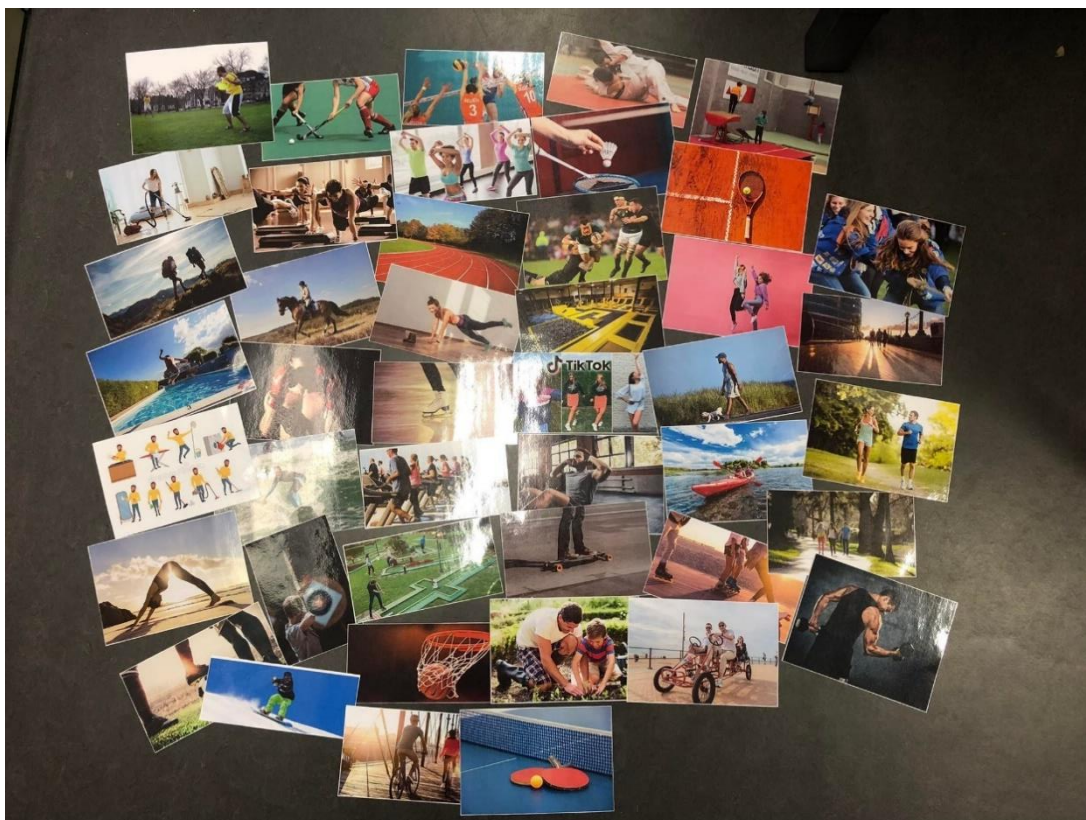

## Activity cards

Supplement: Supplementary file 3 — Additional file 3. Pictures of co-creation session 2. [file 40900_2023_420_MOESM3_ESM.pdf]
