# Supplementary material for: Co-creating an intervention to promote physical activity in adolescents with intellectual disabilities: lessons learned within the Move it, Move ID!-project
Source: Res Involv Engagem. 2023 Mar 19;9:10. doi: 10.1186/s40900-023-00420-x (PMC10024913; doi:10.1186/s40900-023-00420-x)
Supplement: Supplementary file 4 — Additional file 4. Pictures of co-creation session 3. [file 40900_2023_420_MOESM4_ESM.pdf]

SUPPLEMENTARY FILE 4: CO-CREATION SESSION 3

During the second co-creation session, adolescents with ID named all their barriers to engage in physical activity. Between the second and third sessions, the PI then placed all these possible barriers on a micro-meso-macro model (see below, one for each group). This was mainly to have an overview as a research team of the barriers and where/at what level they are located. At the beginning of the third session, the PI then went over this model briefly with the adolescents. This was mainly to repeat all the barriers they had identified in the previous session, and subsequently to check whether any barriers were still missing. Note: the model was not presented to the adolescents all at once, but layer per layer, barrier per barrier. A barrier not mentioned in group A, but only in group B, was not added to the figure of group A.

Model of group A:

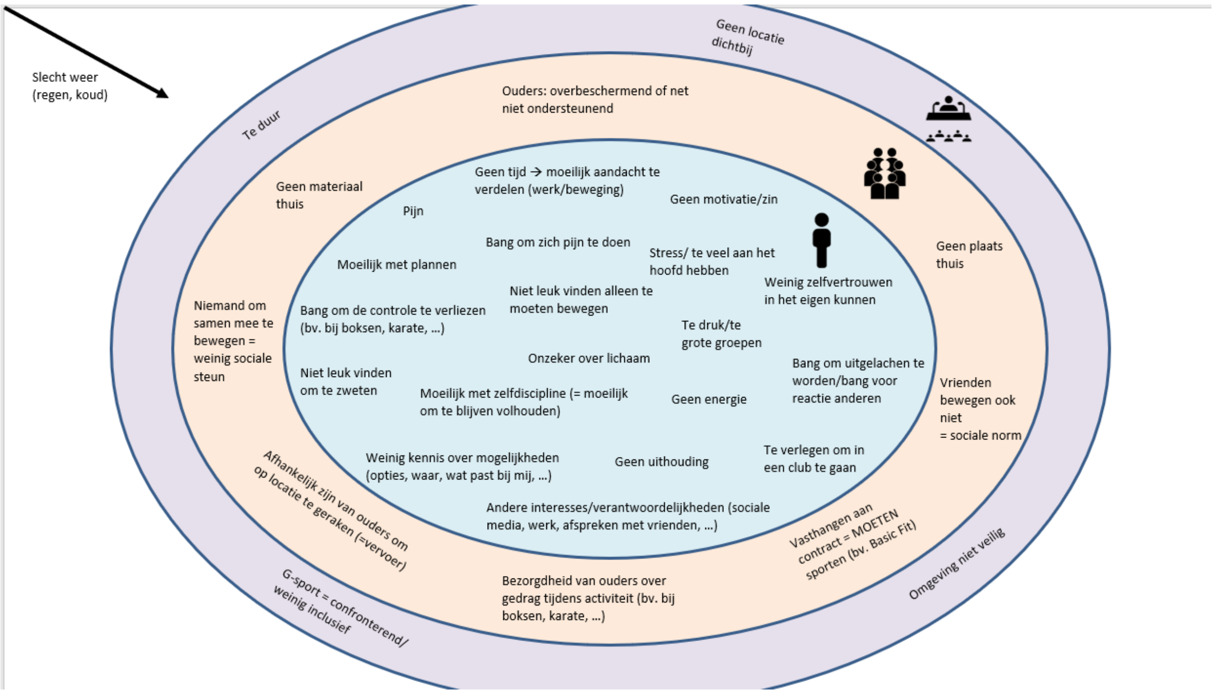

Blue = micro-level; orange = meso-level; purple = macro-level. The arrow shows a barrier that doesn't fit within a level (here: the weather).

Model of group B:

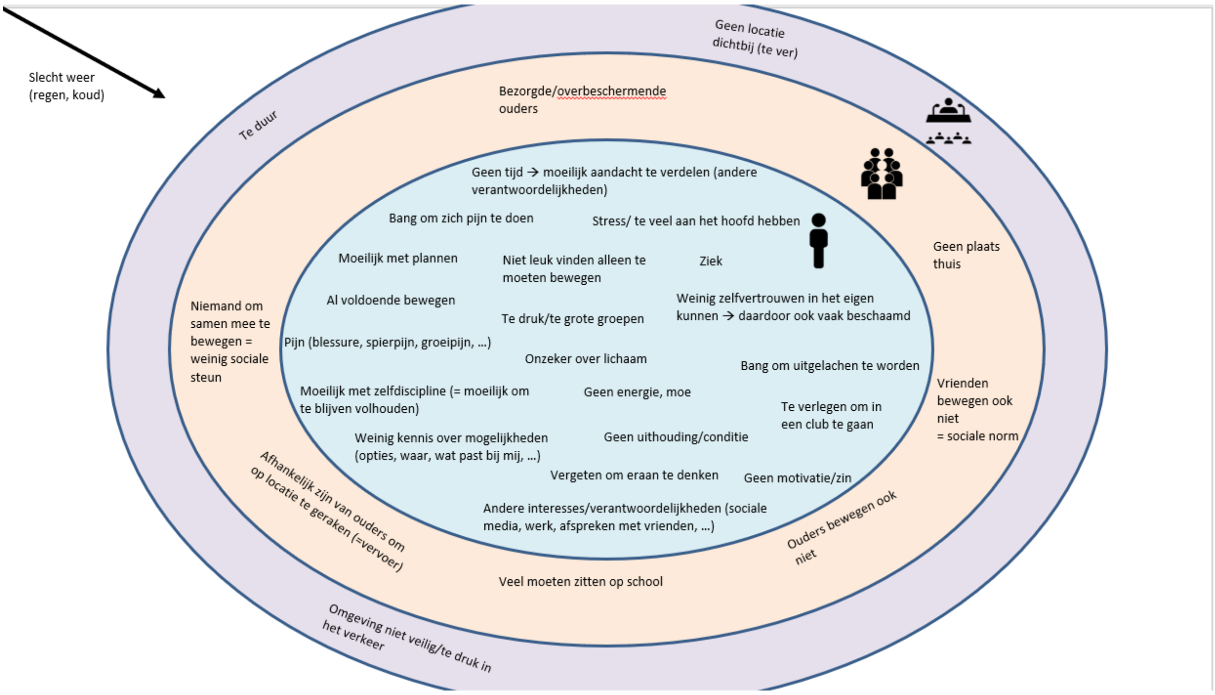

Blue = micro-level; orange = meso-level; purple = macro-level. The arrow shows a barrier that doesn't fit within a level (here: the weather).

After this introduction with the micro-meso-macro-model, adolescents could again creatively engage via voting on intervention goals using stickers, and then also ranking them according to importance.

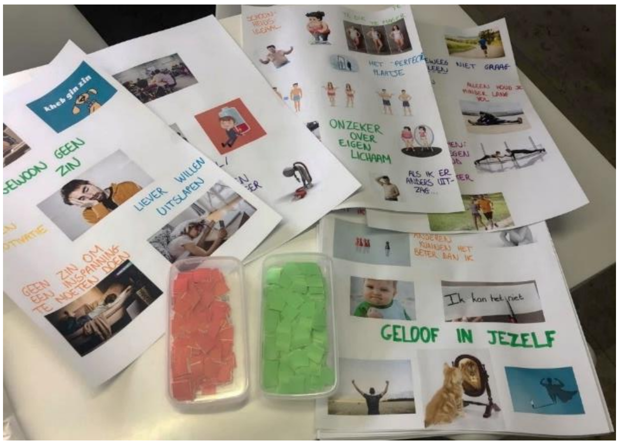

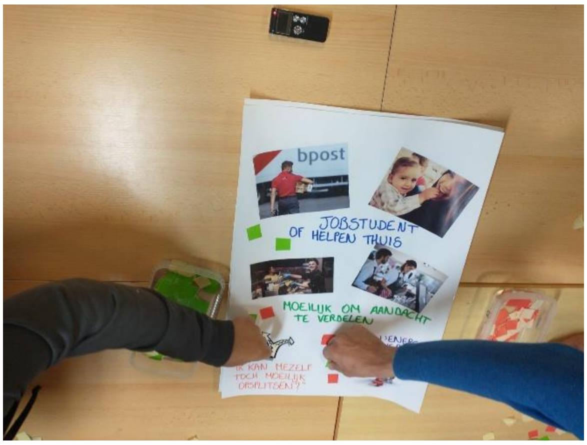

1) Intervention goals adolescents could vote on

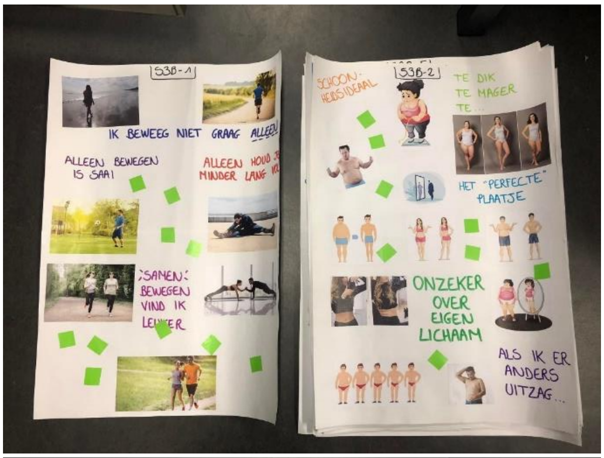
S3B-1
IK BEWEEG NIET GRAAG ALLEEN
ALLEEN BEWEGEN IS SAAI
"SAMEN" BEWEGEN VIND IK LEUKER
S3B-2
SCHOONHEIDSIDEAAL
TE DIK
TE MAGER
HET "PERFECTE" PLAATJE
ONZEKER OVER EIGEN LICHAAM
ALS IK ER ANDERS UITZAG...
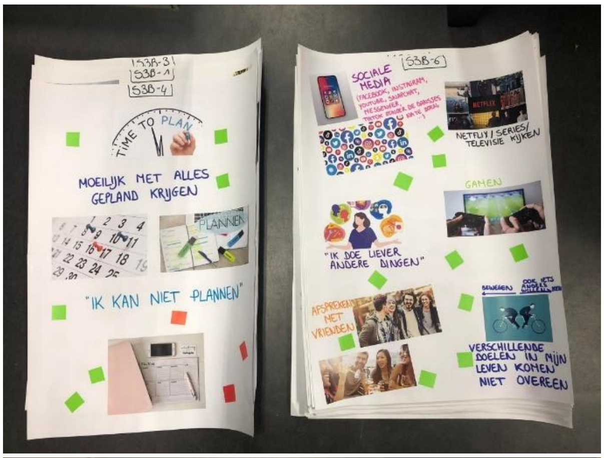
S3B-3
S3B-4
TIME TO PLAN
MOEILIJK MET ALLES GEPLAND KRIJGEN
"IK KAN NIET PLANNEN"
S3B-6
SOCIALE MEDIA
NETFLIX / SERIES / TELEVISIE KIJKEN
GAMEN
"IK DOE LIEVER ANDERE DINGEN"
AFSPREKEN MET VRIENDEN
VERSCHILLENDE DOELEN IN MIJN LEVEN KOMEN NIET OVEREEN
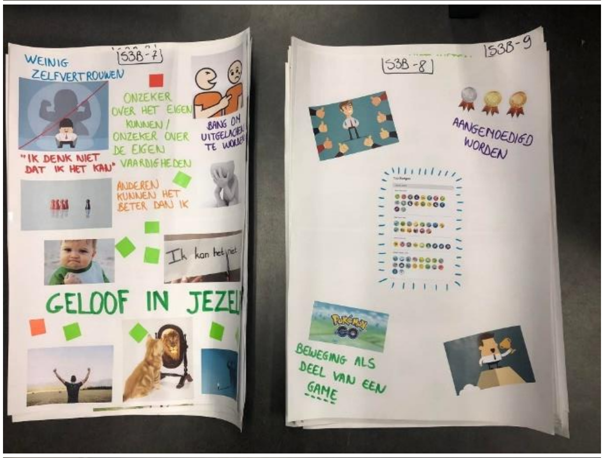
WEINIG ZELFVERTROUWEN
S3B-7
ONZEKER OVER HET EIGEN KUNNEN / ONZEKER OVER DE EIGEN VAARDIGHEDEN
"IK DENK NIET DAT IK HET KAN"
ANDEREN KUNNEN HET BETER DAN IK
Ik kan het niet
GELOOF IN JEZE
S3B-8
S3B-9
AANGEMOEDIGD WORDEN
BEWEGING ALS DEEL VAN EEN GAME
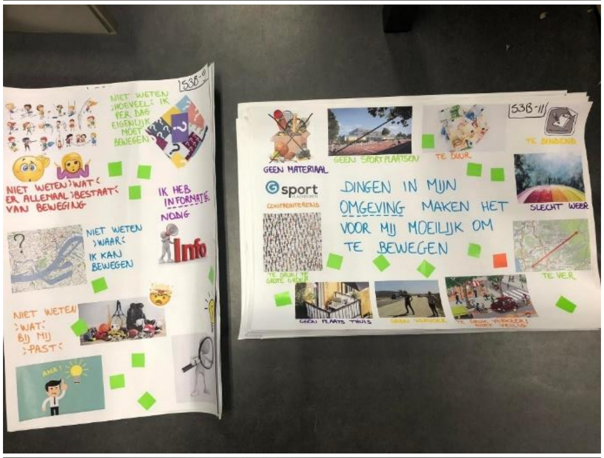
IK HEB INFORMATIE NODIG
Info
S3B-11
GEEN MATERIAAL
GEEN SPORTPLAATSEN
TE DUUR
DINGEN IN MIJN OMGEVING MAKEN HET VOOR MIJ MOEILIJK OM TE BEWEGEN
SLECHT WEER
TE VER
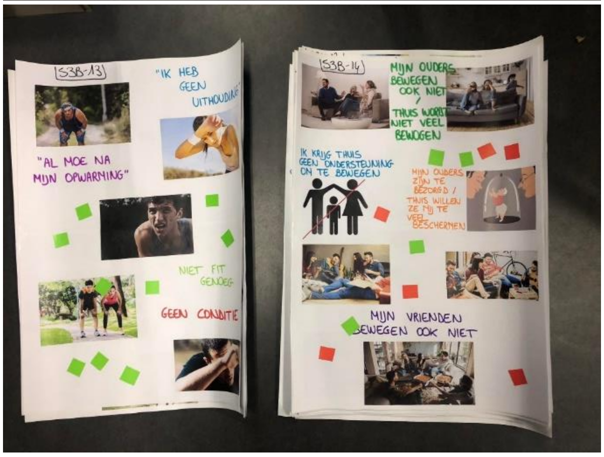
S3B-13
"IK HEB GEEN UITHOUDING"
"AL MOE NA MIJN OPWARMING"
NIET FIT GENOEG
GEEN CONDITIE
S3B-14
MIJN OUDERS BEWEGEN OOK NIET
THUIS WORDT NIET VEEL BEWOGEN
IK KRIJG THUIS GEEN ONDERSTEUNING OM TE BEWEGEN
MIJN VRIENDEN BEWEGEN OOK NIET
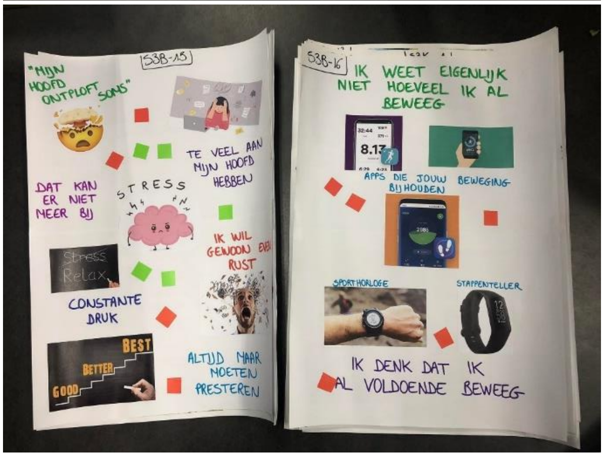
S3B-15
"MIJN HOOFD ONTPLOFT SOMS"
TE VEEL AAN MIJN HOOFD HEBBEN
DAT KAN ER NIET MEER BIJ
STRESS
IK WIL GEWOON EEN RUST
CONSTANTE DRUK
ALTIJD MAAR MOETEN PRESTEREN
S3B-16
IK WEET EIGENLIJK NIET HOEVEEL IK AL BEWEEG
APPS DIE JOUW BEWEGING BIJHOUDEN
SPORTHORLOGE
STAPPENTELLER
IK DENK DAT IK AL VOLDOENDE BEWEEG

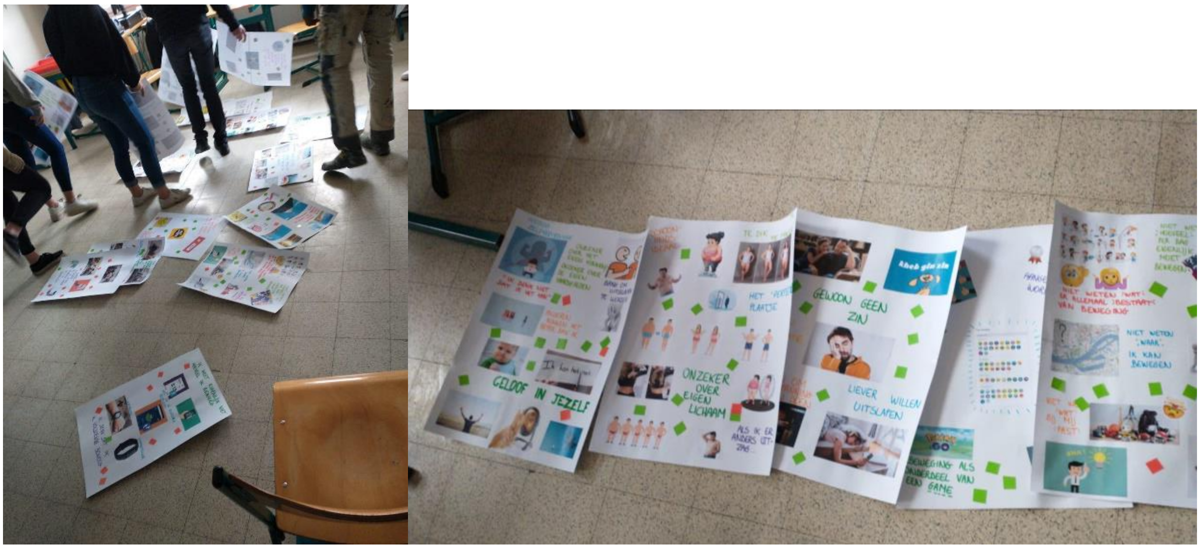

2) Ranking the intervention goals according to importance
