# Supplementary material for: Co-creating an intervention to promote physical activity in adolescents with intellectual disabilities: lessons learned within the Move it, Move ID!-project
Source: Res Involv Engagem. 2023 Mar 19;9:10. doi: 10.1186/s40900-023-00420-x (PMC10024913; doi:10.1186/s40900-023-00420-x)

SUPPLEMENTARY FILE 5: CO-CREATION SESSION 6

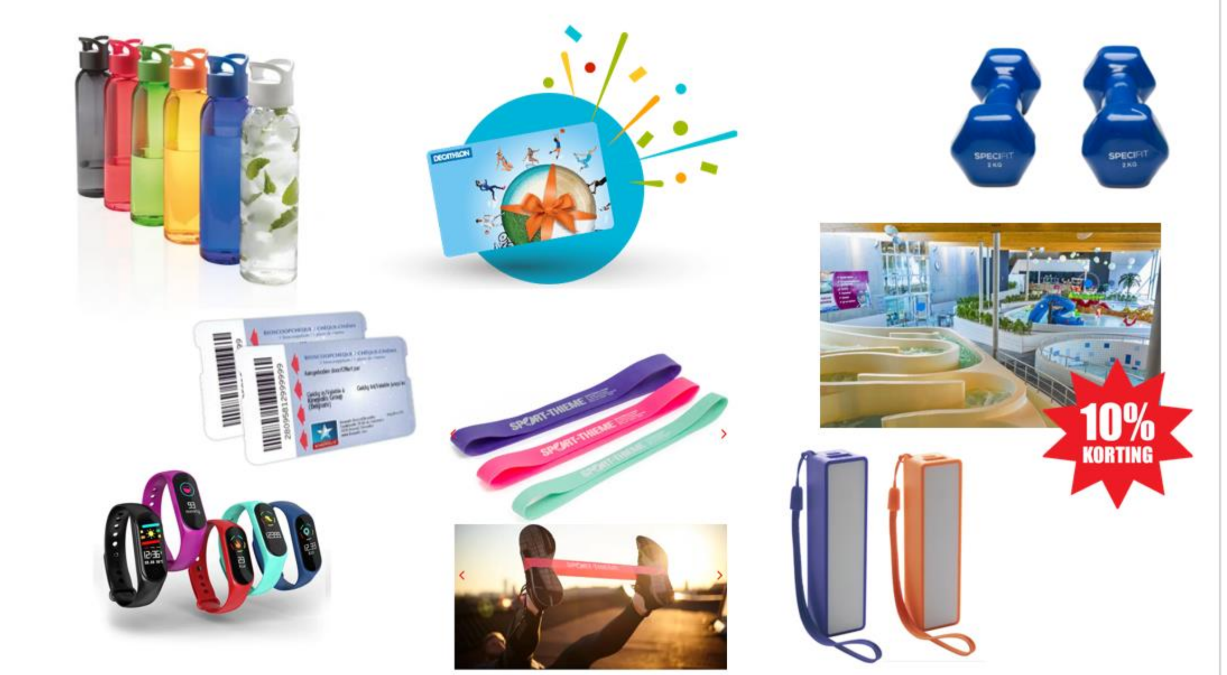

PowerPoint-slide with examples of incentives

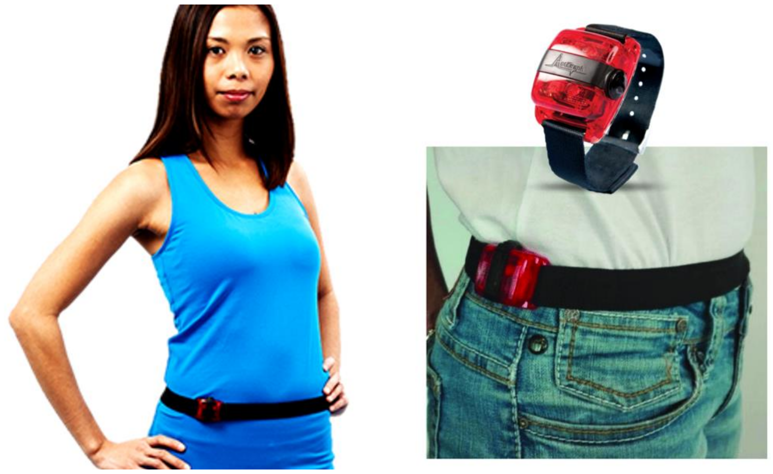

Accelerometers that were brought to the 6th session

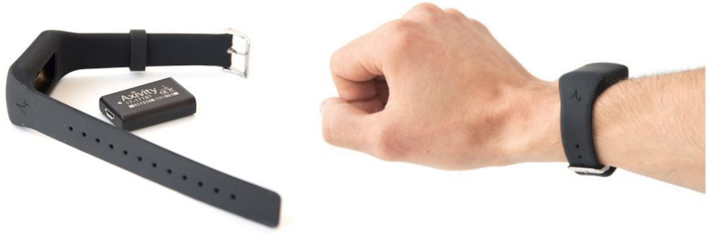

Supplement: Supplementary file 5 — Additional file 5. Pictures of co-creation session 6. [file 40900_2023_420_MOESM5_ESM.pdf]
