# Supplementary material for: Co-creating an intervention to promote physical activity in adolescents with intellectual disabilities: lessons learned within the Move it, Move ID!-project
Source: Res Involv Engagem. 2023 Mar 19;9:10. doi: 10.1186/s40900-023-00420-x (PMC10024913; doi:10.1186/s40900-023-00420-x)
Supplement: Supplementary file 6 — Additional file 6. Reflection form of researchers. [file 40900_2023_420_MOESM6_ESM.pdf]

SUPPLEMENTARY FILE 6: REFLECTION FORM RESEARCHER/ASSISTANT RESEARCHER

Co-creation sessions

Date:

Session nr. en content:

- ☞ What was said? What was not said?
- ☞ Who was involved? Who wasn’t?
- ☞ What was done, what was achieved? What was not done, What was not achieved?
- ☞ Were there strong, outspoken opinions/emotions?
- ☞ What went well? (group level)
- ☞ Which group qualities came to the foreground?
- ☞ How can we bring out the qualities in the group?
- ☞ What can be approved in the group process?
- ☞ What went well? (individual level)
- ☞ What can be approved in the facilitating role of the researcher?
- ☞ Other remarks?

	- -	-	Neutral	+	+ +
Goal of the session was clear					
Entire group participated					
An openness to new ideas and opinions					
Exchange of useful information					
Equal level of commitment					
Everyone could give his/her opinion					
Climate of trust and openness					
Relevant conversations					
Positive atmosphere					
Development of new insights					
Good feeling					
Clear common mission					
Equal influence on decisions (among participants)					
Respectful interactions					
Satisfaction with course and progress					
Use of comprehensible language					
Adolescents learned something new					
Creativity					
Interesting, fascinating					

☞ Remarks on the table?
