# Supplementary material for: Co-creating an intervention to promote physical activity in adolescents with intellectual disabilities: lessons learned within the Move it, Move ID!-project
Source: Res Involv Engagem. 2023 Mar 19;9:10. doi: 10.1186/s40900-023-00420-x (PMC10024913; doi:10.1186/s40900-023-00420-x)
Supplement: Supplementary file 7 — Additional file 7. Description of the results of the adolescents' process evaluations per session and per group. [file 40900_2023_420_MOESM7_ESM.pdf]

SUPPLEMENTARY FILE 7: DESCRIPTION OF THE RESULTS OF ADOLESCENTS' PROCESS EVALUATIONS PER SESSION AND PER GROUP

Table 1: Results of the adolescent process evaluation forms, group A (17-22 year olds)

	Session					
	1	2	3	4 (n=10)	5 (n=9)	6 (n=10)
I was able to express my opinion	Introductory session	Lack of time	Lack of time	+ 10<br>± 0<br>- 0	+ 9<br>± 0<br>- 0	+ 10<br>± 0<br>- 0
Others listened to my opinion	Introductory session	Lack of time	Lack of time	+ 10<br>± 0<br>- 0	+ 9<br>± 0<br>- 0	+ 10<br>± 0<br>- 0
I listened to the opinion of others	Introductory session	Lack of time	Lack of time	+ 10<br>± 0<br>- 0	+ 9<br>± 0<br>- 0	+ 10<br>± 0<br>- 0
I have dared to say everything I wanted to say	Introductory session	Lack of time	Lack of time	+ 9<br>± 1<br>- 0	+ 9<br>± 0<br>- 0	+ 8<br>± 2<br>- 0
I had a good feeling about the conversation	Introductory session	Lack of time	Lack of time	+ 10<br>± 0<br>- 0	+ 9<br>± 0<br>- 0	+ 10<br>± 0<br>- 0
I have learned something	Introductory session	Lack of time	Lack of time	+ 9<br>± 1<br>- 0	+ 9<br>± 0<br>- 0	+ 10<br>± 0<br>- 0
I understood everything that was said	Introductory session	Lack of time	Lack of time	+ 10<br>± 0<br>- 0	+ 9<br>± 0<br>- 0	+ 10<br>± 0<br>- 0
I thought it was interesting	Introductory session	Lack of time	Lack of time	+ 10<br>± 0<br>- 0	+ 9<br>± 0<br>- 0	+ 10<br>± 0<br>- 0
I understand the purpose of this class discussion	Introductory session	Lack of time	Lack of time	+ 10<br>± 0<br>- 0	+ 9<br>± 0<br>- 0	+ 10<br>± 0<br>- 0

+ number of participants that were positive; ± number of participants that were neutral; - number of participants that were negative

Table 2: Results of the adolescent process evaluation forms, group B (14-15 year olds)

	Session					
	1	2 (n = 9)	3 (n=8)	4 (n=9)	5 (n=8)	6 (n=8)
I was able to express my opinion	Introductory session	+ 8<br>± 1<br>- 0	+ 7<br>± 1<br>- 0	+ 8<br>± 1<br>- 0	+ 7<br>± 1<br>- 0	+ 8<br>± 0<br>- 0
Others listened to my opinion	Introductory session	+ 8<br>± 1<br>- 0	+ 8<br>± 0<br>- 0	+ 8<br>± 1<br>- 0	+ 8<br>± 0<br>- 0	+ 8<br>± 0<br>- 0
I listened to the opinion of others	Introductory session	+ 9<br>± 0<br>- 0	+ 7<br>± 1<br>- 0	+ 8<br>± 1<br>- 0	+ 6<br>± 2<br>- 0	+ 8<br>± 0<br>- 0
I have dared to say everything I wanted to say	Introductory session	+ 7<br>± 2<br>- 0	+ 5<br>± 3<br>- 0	+ 7<br>± 1<br>- 1	+ 6<br>± 2<br>- 0	+ 7<br>± 0<br>- 1
I had a good feeling about the conversation	Introductory session	+ 8<br>± 1<br>- 0	+ 5<br>± 3<br>- 0	+ 7<br>± 1<br>- 1	+ 6<br>± 2<br>- 0	+ 8<br>± 0<br>- 0
I have learned something	Introductory session	+ 7<br>± 1<br>- 1	+ 7<br>± 0<br>- 1	+ 8<br>± 0<br>- 1	+ 7<br>± 1<br>- 0	+ 8<br>± 0<br>- 0
I understood everything that was said	Introductory session	+ 5<br>± 4<br>- 0	+ 4<br>± 4<br>- 0	+ 4<br>± 3<br>- 2	+ 5<br>± 3<br>- 0	+ 7<br>± 1<br>- 0
I thought it was interesting	Introductory session	+ 9<br>± 0<br>- 0	+ 7<br>± 1<br>- 0	+ 6<br>± 3<br>- 0	+ 7<br>± 1<br>- 0	+ 8<br>± 0<br>- 0
I understand the purpose of this class discussion	Introductory session	+ 7<br>± 1<br>- 1	+ 7<br>± 1<br>- 0	+ 6<br>± 1<br>- 2	+ 6<br>± 1<br>- 1	+ 8<br>± 0<br>- 0

+ number of participants that were positive; ± number of participants that were neutral; - number of participants that were negative
