# Supplementary material for: Co-creating an intervention to promote physical activity in adolescents with intellectual disabilities: lessons learned within the Move it, Move ID!-project
Source: Res Involv Engagem. 2023 Mar 19;9:10. doi: 10.1186/s40900-023-00420-x (PMC10024913; doi:10.1186/s40900-023-00420-x)
Supplement: Supplementary file 9 — Additional file 9. GRIPP2 Reporting Checklist - Long Form. [file 40900_2023_420_MOESM9_ESM.pdf]

SUPPLEMENTARY FILE 9: GRIPP2 REPORTING CHECKLIST – LONG FORM

Section and topic	Item	Reported on page No
Section 1: Abstract of paper		
1a: Aim	Report the aim of the study	1
1b: Methods	Describe the methods used by which patients and the public were involved	1
1c: Results	Report the impacts and outcomes of PPI in the study	1
1d: Conclusions	Summarise the main conclusions of the study	1
1e: Keywords	Include PPI, “patient and public involvement,” or alternative terms as keywords	1
Section 2: Background to paper		
2a: Definition	Report the definition of PPI used in the study and how it links to comparable studies	2
2b: Theoretical underpinnings	Report the theoretical rationale and any theoretical influences relating to PPI in the study	3<br>*The co-creation sessions were set up according to the Behaviour Change Wheel framework. The COM-B model and TDF framework were central: it was discussed with adolescents with ID which components were relevant to achieve behavioural change (i.e., increased physical activity); how these components were*

Section and topic	Item	Reported on page No
		implemented was done in consultation with them. This whole process is explained in detail in another paper (under review).
2c: Concepts and theory development	Report any conceptual models or influences used in the study	3
Section 3: Aims of paper		
3: Aim	Report the aim of the study	3
Section 4: Methods of paper		
4a: Design	Provide a clear description of methods by which patients and the public were involved	3-6
4b: People involved	Provide a description of patients, carers, and the public involved with the PPI activity in the study	3
4c: Stages of involvement	Report on how PPI is used at different stages of the study	3-6
4d: Level or nature of involvement	Report the level or nature of PPI used at various stages of the study	3-6
Section 5: Capture or measurement of PPI impact		
5a: Qualitative evidence of impact	If applicable, report the methods used to qualitatively explore the impact of PPI in the study	7 *(i.e., in reflection forms of researchers)*
5b: Quantitative evidence of impact	If applicable, report the methods used to quantitatively measure or assess the impact of PPI	4, 7 *(i.e., process evaluation forms of adolescents)*
5c: Robustness of measure	If applicable, report the rigour of the method used to capture or measure the impact of PPI	7

Section and topic	Item	Reported on page No
Section 6: Economic assessment		
6: Economic assessment	If applicable, report the method used for an economic assessment of PPI	NA
Section 7: Study results		
7a: Outcomes of PPI	Report the results of PPI in the study, including both positive and negative outcomes	7-11
7b: Impacts of PPI	Report the positive and negative impacts that PPI has had on the research, the individuals involved (including patients and researchers), and wider impacts	7-11
7c: Context of PPI	Report the influence of any contextual factors that enabled or hindered the process or impact of PPI	7-11
7d: Process of PPI	Report the influence of any process factors, that enabled or hindered the impact of PPI	7-11
7ei: Theory development	Report any conceptual or theoretical development in PPI that have emerged	NA in this paper. *Another paper on this process describes in detail the findings that emerged per phase of the BCW, and how an intervention was developed based on choices made within the BCW. This paper is purely a reflection on the co-creation process and formulates the lessons learned.*
7eii: Theory development	Report evaluation of theoretical models, if any	NA
7f: Measurement	If applicable, report all aspects of instrument development and testing (e.g., validity, reliability, feasibility, acceptability, responsiveness, interpretability, appropriateness, precision)	NA

Section and topic	Item	Reported on page No
7g: Economic assessment	Report any information on the costs or benefit of PPI	14
Section 8: Discussion and conclusions		
8a: Outcomes	Comment on how PPI influenced the study overall. Describe positive and negative effects	12-14
8b: Impacts	Comment on the different impacts of PPI identified in this study and how they contribute to new knowledge	12-14
8c: Definition	Comment on the definition of PPI used (reported in the Background section) and whether or not you would suggest any changes	NA
8d: Theoretical underpinnings	Comment on any way your study adds to the theoretical development of PPI	12
8e: Context	Comment on how context factors influenced PPI in the study	12-14
8f: Process	Comment on how process factors influenced PPI in the study	12-14
8g: Measurement and capture of PPI impact	If applicable, comment on how well PPI impact was evaluated or measured in the study	12, 14
8h: Economic assessment	If applicable, discuss any aspects of the economic cost or benefit of PPI, particularly any suggestions for future economic modelling.	NA
8i: Reflections/critical perspective	Comment critically on the study, reflecting on the things that went well and those that did not, so that others can learn from this study	12-14

PPI=patient and public involvement
